# Interactive learning environment as a source of critical thinking skills for college students

**DOI:** 10.1186/s12909-024-05247-y

**Published:** 2024-03-12

**Authors:** Hao Song, Lianghui Cai

**Affiliations:** 1 School of Civil Commercial and Economic Law, Henan University of Economics and Law, Zhengzhou, China; 2 School of Communication Arts, Wuhan Qingchuan University, WuHan, China

**Keywords:** Critical thinking, Interactive learning environment, Mental efficiency, Mobile learning, Soft skills

## Abstract

**Background:**

The cognitive skills underlying critical thinking include analysis, interpretation, evaluation, explanation, inference, and self-regulation. The study aims to consider the possibility and effectiveness of introducing the mobile game Lumosity: Brain Training into the learning process of first-year Philology students studying at Qiqihar University.

**Methods:**

The sample included 30 volunteers: 15 girls and 15 boys, whose average age was 18.4 years. Before the experiment start, the respondents took a pre-test based on the *Critical Thinking Skills Success* methodology, which was developed by the American scientist Starkey. It was stated that intensive one-month training with the use of the Lumosity premium application in the classroom would improve critical thinking skills.

**Results:**

The pre-test results showed that some respondents had had quite good critical thinking skills before the experiment as the average score was 22.13 out of 30 points. The effectiveness was evaluated using the Student’s t-test for paired samples. It is established that there are significant differences between standard and empirical values (*p* = 0.012).

**Conclusions:**

The research can be of interest to those who study the issue of integrating an interactive learning environment into university and student programs, as well as those who consider critical thinking as a field of scientific knowledge and seek to develop critical thinking skills. The novelty of the study is the fact that students were allowed to use the app only during classes, but the research hypothesis was confirmed. This indicates that an interactive learning environment can be considered as a tool for developing students’ critical thinking skills in the context of limited screen time.

## Introduction

Critical thinking is one of the thinking skills that relates to the cognitive abilities of a person. From a pedagogical perspective, there are two main approaches. The first approach considers critical thinking as a complex combination of knowledge, skills, and predispositions that can be intentionally developed using proper stimuli. According to the second interpretation, critical thinking is a general skill that is more or less impervious to pedagogical impact [1]. Critical thinking skills are among the priorities in education. They contribute to the state’s global economic competitiveness [2]. Even before the pandemic outbreak, critical thinking was studied in the context of creating an interactive learning environment as tablets and smartphones had spread so much that teachers began to actively use ICTs for better and individualized learning [3]. Critical thinking as a mental process is characterized by objectivity, perseverance, and involvement when a person has to face a problem, an opposing opinion, or disagreement. Carefully designed interactive learning settings create favorable conditions for reflection and critical thinking, promote the development of higher education through better student involvement, popularize online learning, and contribute to effective social interaction in the conditions of reduced face-to-face contacts [4]. The relevance of the research is due to the fact that mobile applications integrated into university programs can improve students’ critical thinking skills and create prerequisites for media literacy formation. The gadget’s screen can provide space and time to improve thinking skills by engaging users in a systematic and sustained critical discourse based on interactive tasks [5]. Moreover, information technologies enrich the mental experience of recipients and contribute to the development of reflexive skills needed for critical thinking.

### Literature review

Critical thinking was analyzed within the framework of studying chemistry and physics using mobile learning tools [6, 7]. The benefits of integrating gadgets were noted as both students’ critical thinking skills and academic performance improved. Critical thinking improvement is often classified as the most important goal of formal education as the ability to think critically ensures success while teaching critical thinking skills is sometimes only part of a higher education curriculum [8]. Blended learning is often used for the development of critical thinking in the student environment. This approach is mainly based on a combination of hand-on classes, classroom discussions, online discussions, interactive simulators, and various assessment forms that involve and instruct students, as well as ensure a productive asynchronous interaction with the teacher [9]. It is shown that critical thinking, as well as academic performance, can be improved by using educational games such as Role Play Games [10]. Currently, mobile software is considered progressive in terms of developing critical thinking skills. This is due to the fact that students adapt faster to any learning environment; they get the opportunity to actively participate in the learning process, solve interesting theoretical and practical problems based on critical thinking [11]. The analysis of pedagogical impact characteristics shows that effective critical thinking development is determined by many factors. First of all, these are personal (student’s learning style and motivation); methodological (methods, pedagogical techniques, duration of classes, feedback) and contextual (classroom atmosphere, incentive system) factors [12]. It was concluded that gamification should be considered as a valuable pedagogical tool that encourages users to master educational systems and demonstrate a higher engagement rate [13]. Critical thinking as an individual’s intellectual ability to analyze a problem using raw data and various solution strategies is a skill that can be improved relatively easily by appropriate cognitive stimulation [14]. Since the Enlightenment, critical thinking was seen as the core of scientific innovation. However, after the Bologna Process, this consensus disappeared as other goals such as efficiency, professional relevance, mobility, result orientation, and competence came to the fore [15]. The promotion of critical thinking in higher education has been increasingly requested by society. Thus, for example, in 2017, the German authorities decided to introduce critical thinking into university curricula [16]. Moreover, in order to determine the optimal concept for a particular educational institution, it is necessary to analyze the framework conditions of pedagogical practice; set learning goals to develop thinking; promote critical thinking in university education; create a proper learning atmosphere; implement and adapt a program for critical thinking development, which can also rely on an interactive platform. Critical thinking as a form of reasoning can represent “good thinking” and superior “higher-order” thinking. However, there is a need for a transcultural approach to critical thinking as culturally specific ways of thinking and an assessment system that attaches particular importance to certain forms of reasoning can be obtained only from the synthesis of social imagination and thinking, which are monolingual. For example, a focus on tolerance, common in Western Europe, may not dovetail with the worldview of a student who came from Asia [17]. In the context of an interactive learning environment, computational and computer skills that have common features with critical thinking are also studied. These include the ability to solve a problem in an innovative way; the ability to exactly determine what should be derived from a problem in order to find an optimal solution; the ability to effectively reflect on the most important elements of the problem and exclude irrelevant factors from the mental canvas [18]. Mobile learning has been identified as a strategy that can increase students’ thinking skills of the highest order [19]. However, mobile technology effectiveness is also determined by the learning process, which takes place along with the use of an interactive learning environment [3]. At the same time, a strong foundation in the form of critical thinking means that students will be able to solve higher-order issues that require analysis, comparison and evaluation earlier and with greater readiness. Moreover, students’ ability to assess information reliability during its teaching rather than after the fact can make academic classes more productive [20]. Effective knowledge can be constructed by involving recipients in meaningful contexts of social interaction and life experience. Unfortunately, traditional pedagogy has failed to ensure this in order to develop the active role of students in education, improve their thinking skills and cognitive abilities, including critical thinking [21]. Creativity as a thinking skill is most often compared to critical thinking as it is a higher-order mental skill that includes the ability to rely on lower-order thinking skills, generate innovative ideas, combine parts to create a whole, as well as plan the creation and development of new products in different spheres of life. Meanwhile, critical thinking has many special characteristics as it is a specific process of clear thinking and rational analysis of facts and events [22].

The critical analysis of the literature reveals a consistent theme: the integration of mobile learning tools in education significantly enhances critical thinking skills. Studies by Astuti et al. [6] and Cahyana et al. [7] in the fields of chemistry and physics demonstrate the positive impact of mobile learning tools on both critical thinking and academic performance. This synergy of technology and education is further echoed in the findings of Leal et al. [11]. The authors note the rapid adaptation and active engagement of students in learning environments augmented by mobile software. This adaptation is not only confined to the absorption of knowledge but extends to the application of critical thinking in solving complex theoretical and practical problems. Similarly, the work of Lorencová et al. [12] highlights the multifaceted nature of critical thinking development. In this context, the researchers emphasize the importance of personal, methodological, and contextual factors in pedagogical practices. The above studies collectively suggest that the integration of technology in education, especially mobile learning tools, is a critical factor in enhancing critical thinking skills.

Furthermore, the synthesis of the results underscores the transformative role of innovative teaching methods like gamification and blended learning in fostering critical thinking. Borglum [9] and Rasyid et al. [10] illustrate how educational games and blended learning approaches, respectively, significantly facilitate the development of critical thinking skills. These methods, which combine traditional and digital pedagogical techniques, engage students more effectively, as well as provide diverse platforms for them to apply critical thinking.

### Problem statement

Mobile games are popular leisure activities that can be used to improve mental functions. This suggests that users from all over the world can develop their problem-solving skills while enjoying the game. Numerous mobile games, including adventures, puzzles, car racing, shooters, and sports, can contribute to the cognitive development of a person, preparing them for faster decision-making, stimulating the brain, and rewarding successful task completion [18]. Critical thinking can be indirectly developed by video games offering veiled exercises in strategy, tactics, and problem-thinking, as seen in games like Plague Inc. or Minecraft. Furthermore, there are interactive mobile applications specifically aimed at developing critical thinking skills, such as Logic Master, Can You Escape, Skillz, Brain games, Brainiton, Unblock me, and others [19]. The analysis of the existing literature [23–27] shows that the discussion mainly focuses on assessing the overall impact of mobile games on cognitive development, rather than on critical thinking skills. However, the latter is no less important and this study aims to address this gap. Therefore, it explores how an interactive learning environment created with the mobile game Lumosity: Brain Training can develop critical thinking skills in first-year college students.

The research aims to empirically study the effectiveness of digital software introduction to improve critical thinking skills in the student environment. The following tasks have been set: formation of an experimental group to test the hypothesis; selection and execution of optimal software with a focus on age relevance; preliminary assessment of respondents’ critical thinking skills; and comparison of initial skills with those formed after repeated interaction with an application that ensures an interactive learning environment.

Lumosity: Brain Training presents a unique approach to cognitive development. This application focuses on enhancing various cognitive domains, such as memory, attention, flexibility, speed, and problem-solving. Based on neuropsychological tasks, the app provides a range of games that adapt to the user’s performance, ensuring a challenging and personalized experience. It tracks progress over time, offering users valuable feedback on their cognitive abilities and areas for improvement. The gamified nature of Lumosity increases engagement and motivation, which are crucial for consistent use and cognitive improvement.

## Methods and materials

The study primarily utilizes the Critical Thinking Skills Success Technique developed by American researcher Starkey [28]. This method was chosen due to its structure, which consists of a Pretest and a Post-test. The Pretest is designed to determine the initial level of the respondent’s critical thinking skills. It comprises 30 questions, each with four answer options, where only one is correct. Notably, there is no time limit for completing the test, and respondents are awarded 1 point for each correct answer. The Post-test is structured similarly to the Pretest, facilitating a comparison of results after the experimental part of the study. This method is intended for individuals over 16 years of age [29]. The questions from the test evaluate various aspects of critical thinking (problem solving, decision making, logical reasoning, analysis and evaluation of statements). Tasks range from evaluating arguments and conclusions to identifying logical errors and making evidence-based judgments [29].

**Fig. 1:**
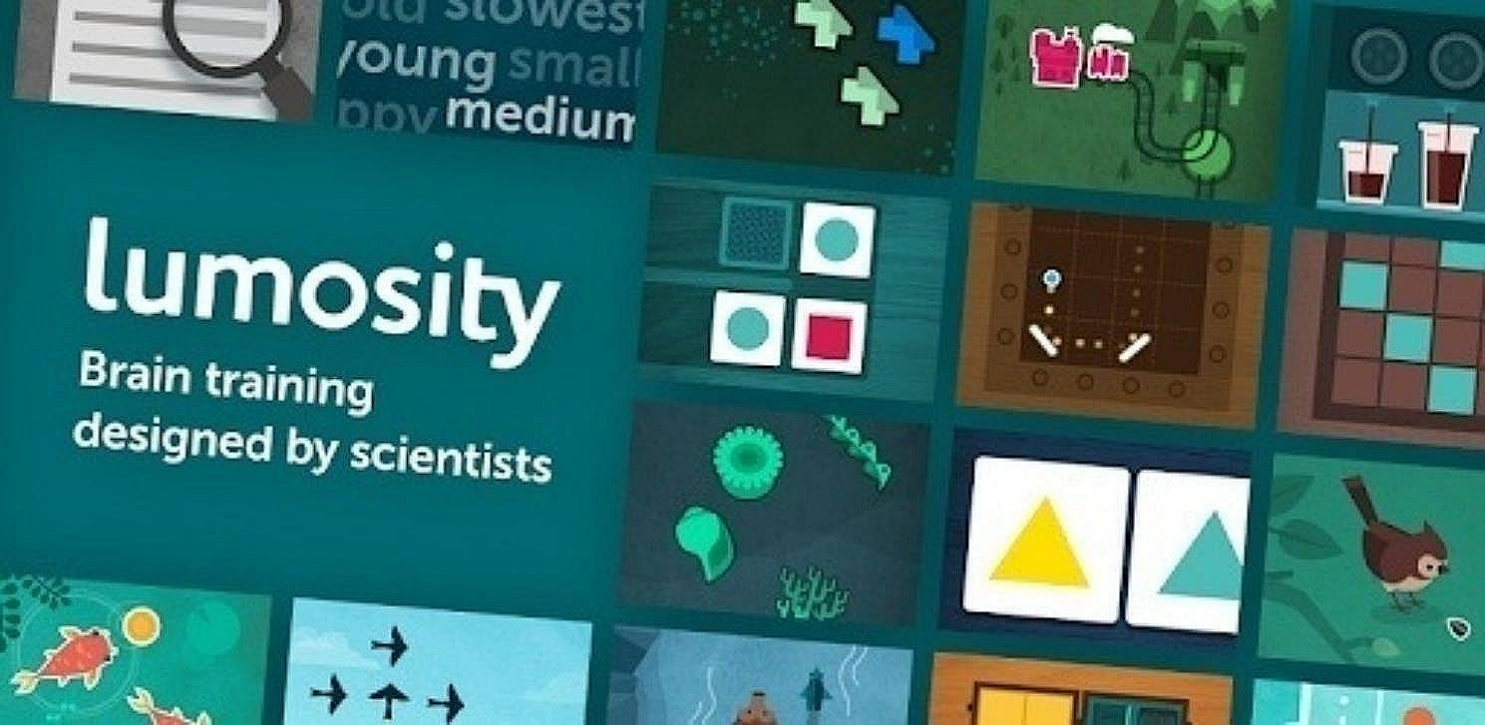
Lumosity: brain training interface

The interactivity of the learning environment was ensured through the use of the Lumosity: BrainTraining mobile application. This software primarily includes games that train memory, speed, flexibility of thinking, and problem-solving skills, which are crucial components of critical thinking. According to the developers, the game is based on cognitive, neuropsychological, and experimental tasks that are transformed into interactive games and puzzles. The study employs the Lumosity Premium subscription, which offers an individualized training program, unlimited access to games, detailed progress information, and recommendations for improving game accuracy, speed, and strategy. The application’s interface is illustrated in Fig. 1.

It is important to note that the application under study does not specialize in critical thinking per se but provides comprehensive cognitive stimulation. This choice was made due to the limited availability of market options. Apps for critical thinking development on Google Play generally have low interactivity and tend to focus more on disseminating information about critical thinking and its improvement methods, such as Critical Thinking, Critical Thinking Insight, Learn Critical Thinking Offline Guide, and others. On the App Store platform, there is only one app specifically aimed at critical thinking development for elementary schoolchildren, the Critical Thinking Activities game by Ventura Educational Systems.

The research received no support from LumosLabs, as they were not a party involved in the study. The monthly subscription for each member of the experimental group was funded by Qiqihar University. There were no material or non-material benefits involved, and the choice of the premium version was purely for the research purpose of evaluating the effectiveness of a short, intensive use of this type of mobile application.

After one month of training, the experimental group was administered a one-factor Post-test consisting of 30 questions to reflect progress in critical thinking. A dependent samples t-test was used as the statistical method to compare the means from the two related groups.

This research also included a pilot study. The objective of the pilot study was to test the feasibility of the methodologies and the effectiveness of the mobile applications in a smaller, controlled setting before the full-scale implementation. This preliminary phase involved a select group of participants exposed to the same mobile applications and testing procedures as planned for the main study. The pilot study allowed for an initial assessment of the research design, the suitability of the selected mobile applications for enhancing critical thinking, and the adequacy of the testing instruments. It also provided an opportunity to refine the research methodology based on the feedback and observations gathered during this phase. The insights gained from the pilot study were instrumental in making necessary adjustments to ensure the validity and reliability of the main study. The pilot study helped determine the optimal frequency and duration of sessions in Lumosity: Brain Training. Consequently, it was possible to maximize cognitive benefits without causing fatigue or disinterest among participants. As a result, the psychometric tool did not cause misunderstanding on the part of participants.

### Participants

The study involved 60 first-year student volunteers studying at the Faculty of Philology of Qiqihar University (the average age of the respondents was 18.4 years) divided into two groups of equal size and composition, experimental and control. The sample was representative in terms of biological gender (15 males and 15 females in both groups).

Considering the number of students in the faculty as a general sample, the allowable sampling error does not exceed *p* = 4.82, which allows us to state that the sample is representative of the given educational institution and the age of the participants. The choice of first-year students is due to the minimal development of critical thinking skills that are instilled in the course of university education, which allows more accurate judgment of the results of the impact of the application. In addition, the assessed impact is planned to be focused primarily on high school students and first-year students.

Anyone could join the study. After taking the pretest, the experimental group got access to a monthly premium subscription of the *Lumosity: Brain Training* app and used it exclusively in the classroom. The principle of voluntariness ensured relatively high motivation and academic discipline. The respondents were interested in the topic and at the very beginning gave their written consent not to play the game outside the classroom and not to use other cognitive stimulations aimed at improving critical thinking skills. This refers to applications similar in content, video games, online courses, training sessions, books. They were also given a possibility of leaving the group at any stage without prior approval and sanctions.

### Research design

This study involved 60 first-year philology students from Qiqihar University. The participants were randomly assigned to either the control or the experimental group. This randomization process was a crucial step in the research design. It minimized any systematic differences between the groups. By allocating students randomly, the study ensured that each group was comparable in terms of various potential confounding variables, such as prior knowledge, cognitive abilities, and learning styles.

Both classes involved in the study were taught by the same teacher. This arrangement was critical for consistency in the delivery of course content and teaching methodology. With a single educator responsible for both the control and the experimental groups, it was possible to effectively monitor variables related to teaching style, instructor expertise, and interaction dynamics.

In the first stage of the study, the respondents performed the Critical Thinking Skills Success pre-test to assess their critical thinking skills for both the experimental and control groups. Participants in the control group did not receive any intervention, did not work with the described software. Both groups continued to follow the same university curriculum; no differences in the methods or means of teaching, as well as special means of developing critical thinking, other than those described here, were used in both groups. Participants in the experimental group were divided into micro-groups of 10 people. The respondents used the application for 30 min 5 days a week. The teacher was asked to encourage students at the beginning of the lesson and provide feedback to help them summarize what games they played and what results were achieved. A total of 20 classes were conducted. In the third week of the experiment, the respondents were given an opportunity to play in pairs. This created dynamics and revived the process through introducing the element of competition. At the same time, the micro-group leaders did not focus on the competitive strategy reinforcement but rather on introspection and cooperation. The research participants took advantage of the premium subscription and chose the most interesting games; they shared strategies to solve the problem and tracked their LPI, that is the LPIs for speed of thinking, memory, attention, flexibility of thinking, problem solving. After 1 month, the philology students completed a post-test duplicating the one they took before the experiment. This made it possible to assess critical thinking before and after the mobile game introduction.

### Data analysis

To study the impact of the applied intervention, a comparison of the results of post-tests and pre-tests of the control and experimental groups was used, as well as a comparison of the results of post-tests for the control and experimental groups. The Student’s *t*-test method was used with the strength of the effect tested according to Cohen’s *d* method. The use of the Student’s t-test in the article is due to the focus on comparing the average values of two independent groups. This tool can show if there is a statistically significant difference between them.

To verify that the sample and pre-experimental design met the requirements of parametric research methods, the results of the pre-test in both groups were checked by the Shapiro-Wilk method. Results B = 0.894 for the control group and B = 0.901 for the experimental group, which indicates that the samples were obtained with a distribution as close to normal as possible.

### Statistical processing

Statistical data were processed in SPSS Statistics 23. The study relies on the Student’s t-test, which assesses the software impact on the cognitive abilities of young people in the field of critical thinking.

### Research limitations

Initially, the Critical Thinking Skills Success methodology was developed within the framework of the course by Starkey (Critical thinking skills Success in 20 min a day) consisting of 20 lessons devoted to certain critical thinking aspects. For example, problem recognition, finding resources, inductive reasoning, and others. Today in the scientific community this test is used without reference to the textbook by Starkey [30]. However, this can be attributed to limitations. The relatively short duration (1 month) and high frequency of software use (20 classes/4 weeks) might have also affected the results of the study. From the neuropsychology perspective, the brain of first-year students is quite flexible but it takes time to consolidate positive changes. The respondents’ age also limits the possibilities for the legitimate retransmission of experimental trends. For example, the brain of masters functions differently due to age-related changes and higher rigidity, which is a natural ontogenesis part. Although masters, as well as first-year students, can be attributed to the youth. When testing the hypothesis about the possibility of purposeful critical thinking development in the classroom, the use of software was limited by the university. This did not allow the experimental group students to use the application in their free time and additionally develop basic thinking functions and critical thinking skills.

### Ethical issues

This study complies with basic ethical standards. For example, the principle of the research benefits was fully implemented. Thus, the participants not only had an opportunity to test their critical thinking skills for free but also could develop these skills by taking advantage of the premium subscription sponsored by Qiqihar University. The fair sample selection principle was also partially implemented as first-year students studying at the Faculty of Philology were enrolled in the study as volunteers willing to join the experiment. However, the sample size was limited. It involved one faculty and considered one year of study. Moreover, 15 young men and 15 young women were included into the group. This means that some students could have been discriminated against because of their biological sex. At the same time, from the very beginning, the respondents knew that they were involved in the study evaluating the effectiveness of mobile applications in the development of critical thinking skills. The principle of respect for the respondent personality and autonomy was ensured at the research design stage as the statutory document contained an option of refusing participation in the experiment at any stage. The group involved only adult volunteers who showed interest in the assessment and development of critical thinking. There was no risk to the physical and mental health of the first-year students. Moreover, prior to the beginning of the empirical experiment, informed consent forms containing data on research participation, research duration, and confidentiality were signed.

## Results

The primary data analysis shows that before the start of the study, first-year students were relatively competent in the field of critical thinking. The pre-test mean was 22.13 out of 30 points, and the range was characterized by wide variability. For example, the minimum score was 14 points and the maximum score was 28 points. The primary distribution data are presented in Table 1.

**Table 1:** Primary data analysis in experimental group

	N	Minimum	Maximum	Mean	Standard deviation
Pretest	30	14	28	22.13	4.485
Post-test	30	19	30	24.50	3.288

It must be said that an interactive learning environment contributed to the development of critical thinking in the experimental group as the lower post-test limit was 19 points, and the upper limit was 30. Moreover, the mean increased up to 24.50 points. This means that the app use really stimulated brain activity. However, there were cases when the post-test result was lower. This may be due to a weakening interest in the study, situational factors, fatigue (intensive classes as a limiting parameter), arrogance and the fact that the post-test contains vocabulary from the Starkey methodology, for example, the names of logical errors that were not displayed in the pre-test [28]. In terms of statistical significance, the results were compared using the Student’s t-test for related samples. The analysis showed that there were significant differences between the standard and empirical values (*p* = 0.012) at the acceptable level of *p* = 0.05. The data are shown in Table 2.

**Table 2:** The results of using the Lumosity: Brain Training impact on students’ critical thinking skills in the experimental group compared with the control group (Student’s t-test)

		Mean	SD	df	t-criterion	p	Cohen’s d
Pre-test	Exp.	22.13	4.485	29	-2.679	0.012	0.88
	Cont.	14.92	3.809	29	2.994	0.891	0.29
Post-test	Exp.	24.50	3.288	29	2.758	0.028*	1.22*
	Cont.	15.12	3.679	29			

*comparison of post-test results for the control and experimental groups

As follows from the results of comparing the pre-test and post-test results for the control group, the changes in the level of critical thinking in this group are not statistically significant (*p* = 0.891) and the strength of the effect can be ignored (0.29). At the same time, in the experimental group, the apparent increase in mean scores (Table 1) from 22.13 to 24.5 is statistically significant (*p* = 0.012) and has a noticeable effect size (0.88). Comparison of post-test results for the experimental and control groups is also statistically significant (*p* = 0.028) and has an apparent effect size (1.22), indicating a large change and a strong influence of the studied variable on critical thinking in the experimental group.

As p = < 0.05, it can be concluded that there are statistically significant differences in critical thinking levels before and after the premium app subscription use. Moreover, given that the t-criterion value is negative (-2.679), a statistically significant increase in critical thinking after intensive one-month software use can be noted. This indicates the effective impact of the cognitive stimulation on the recipients’ brains. The research hypothesis was confirmed. This allows us to believe that the construction of an interactive environment in the classroom has a positive effect on the development of mental skills, which include critical thinking.

The reported effect sizes provide a profound understanding of the practical implications of the research findings. It is especially relevant in terms of the impact of interactive learning environments on critical thinking skills among first-year students. The large effect size of 0.88 in the experimental group indicates a substantial improvement in critical thinking skills due to the use of the interactive mobile application. Thus, the average performance of students in the experimental group was markedly better compared to the control group. This fact suggests the effectiveness of the intervention.

Furthermore, an even more significant effect size of 1.22 in the comparison of post-test results between the experimental and control groups underscores the efficacy of the intervention. Such a large effect size implies that students using the interactive learning tool significantly improved their critical thinking skills compared to their initial abilities. Moreover, they considerably outperformed their peers in the control group. This finding demonstrates that the interactive learning environment was effective and superior to traditional teaching methods or non-interactive learning environments in enhancing critical thinking skills.

On the other hand, in the control group, there was no significant change in critical thinking levels. The small effect size observed in this group emphasizes the limited development of these skills without interactive intervention. This contrast further validates the value of the interactive tools used in the experimental group.

In essence, the effect sizes from the study provide compelling evidence of the practical effectiveness of interactive learning environments in developing critical thinking skills. The results suggest that within educational settings, interactive mobile applications can substantially enhance critical thinking abilities among students. Thus, the findings offer important implications for educational practices and the potential of technology-enhanced learning in fostering essential cognitive skills.

This indicates the statistical significance of the results. A p-value below 0.05 confirms the hypothesis that the intervention (with an interactive mobile application) developed critical thinking skills. The effect size, quantified as 0.88 and 1.22, is considered large based on Cohen’s d-test, where 0.2 represents a small effect, 0.5 is an average effect, and 0.8 is a large effect. Therefore, the use of interactive mobile applications is reasonable for the development of critical thinking skills among college students. This conclusion has implications for the educational environment. The integration of technological learning tools into curricula can be a way to develop critical thinking skills in different parts of the world. In addition, interactive learning has the potential to significantly affect mental abilities.

## Discussion

The results of the empirical study confirm the earlier conclusions [31–33] that indicate a positive correlation between the use of technology, especially interactive learning environments, and the level of critical thinking skills. The improvement observed in the experimental group compared to the control group highlights the effectiveness of educational technologies in the development of cognitive skills. This finding is consistent with the conclusions of other authors [8, 10, 34, 35]. Previous studies have also substantiated the positive role of digital tools in learning outcomes. The article, among other things, expands the dialogue on the need to adapt educational methods to the digital landscape [36, 37].

The results continue the debate about the longevity and sustainability of cognitive improvements after mobile gaming. Therefore, the study prompts further research into the long-term effects of constant use of brain training applications. This aspect echoes the manuscripts that have studied the dynamics of interactive intervention on cognitive functions [33, 38, 39].

The study also opens up opportunities for future research, including comparative analysis of different demographic groups and educational contexts. Additional studies can ensure the universality of the observed benefits. This approach calls for a more detailed understanding of how different populations interact with and benefit from educational technologies. At the same time, the potential of applications such as Lumosity: Brain Training reveals new prospects for personalized education, as was also discussed earlier [6, 12, 14, 40].

In this section, it is also necessary to address the aspect of limitations to make the perception of the results more holistic. The study focuses on a specific demographic group from Qiqihar University. This fact limits the possibility of generalization since the results may not be applicable to a wider and more diverse population group. In addition, the gender distribution was not proportional, which may increase the bias of the article. The duration of the study and the intensity of software use are factors that could have also affected the results. The limited sample size of only 30 volunteers may not reflect the broader group of first-year philology students. The design of the study implied the limited use of the application in the classroom. However, there are potential differences in the use of the application in real life. The measurement of critical thinking skills may depend on situational factors, which may also have an impact. These limitations define the range of future research to fully assess the educational potential of mobile applications such as Lumosity: Brain Training for the development of critical thinking skills in college students.

## Conclusions

In the course of the theoretical and empirical research, it was found out that mobile applications aimed at the development of brain activities can improve students’ critical thinking skills. The interactive learning environment stimulates brain activity, helps learners develop memory, speed, flexibility of thinking, and problem-solving skills. The research hypothesis has been confirmed: one-month training based on the Lumosity premium subscription has improved the critical thinking skills of first-year students studying at the Faculty of Philology. The sample consisted of 30 volunteers who, prior to the experiment, took the Critical Thinking Skills Success pre-test in order to assess their initial level of critical thinking. Next, they participated in a four-week training course containing 20 classes of 30 min. At this time, the students had an opportunity to play those games that attracted them the most. When choosing a game, the learners could rely on an individual plan available within the monthly subscription or ignore the program instructions. It is important to note that the informed consent assumed that the experimental group would not use the application outside the classroom or try to improve critical thinking skills through online courses, similar applications, books. This was done in order to assess the potential of the app use in the classroom. In the second week, the students were allowed to team up and play together to increase engagement and maintain interest. At the end of the premium subscription, the experimental group took the Critical Thinking Skills Success post-test. It has been empirically proved that the Lumosity premium subscription has contributed to an increase in the students’ critical thinking skills. This indicates that critical thinking can be developed in students directly within the academic program without relying on the extracurricular use of brain training games, in which students may quickly lose interest. Critical thinking will be more effectively developed when it is taught within media literacy, sociology, and psychology disciplines and has a relatively long and purposeful impact on the student’s brain activity. The practical value of the research lies in the fact that brain-training applications can now be integrated into the learning process not only in the classroom but also in the extracurricular aspect. For example, it can be a campus competition allowing students to demonstrate progress in the brain-training game and have fun. The scientific value of the research lies in the fact that an empirical two-stage study was conducted to compare the development of critical thinking before and after cognitive stimulation implemented with the help of the application. This promotes the discussion about the mobile software benefits in higher education and motivates app developers to expand the range of marketing proposals aimed at developing critical thinking in adolescents and young people. The research results can be used to develop mathematics, journalism, sociology, linguistics, and psychology curricula and to form the basis of a media literacy course. This study can be of interest to those who are interested in an interactive learning environment or seek to develop critical thinking skills. The research perspective is to evaluate the effectiveness of the Lumosity: Brain Training app based on various samples consisting of other students but not of volunteers. Moreover, it would be useful to study the program for technical students and compare the effectiveness of one-month and three-month uses of the game.

## Data Availability

The datasets used and/or analysed during the current study are available from the corresponding author (Lianghui Cai, lianghuicai6@gmx.com) on reasonable request.
